# Residue Analysis and Assessment of the Risk of Dietary Exposure to Domoic Acid in Shellfish from the Coastal Areas of China

**DOI:** 10.3390/toxins14120862

**Published:** 2022-12-08

**Authors:** Guanchao Zheng, Haiyan Wu, Hanyu Che, Xiaokang Li, Zhihua Zhang, Jixing Peng, Mengmeng Guo, Zhijun Tan

**Affiliations:** 1 Key Laboratory of Testing and Evaluation for Aquatic Product Safety and Quality, Ministry of Agriculture and Rural Affairs, Yellow Sea Fisheries Research Institute, Chinese Academy of Fishery Sciences, Qingdao 266071, China; 2 College of Food Science and Engineering, Ocean University of China, Qingdao 266003, China; 3 College of Food Science and Technology, Shanghai Ocean University, Shanghai 201306, China; 4 Hebei Province Aquatic Products Quality Inspection and Testing Station, Shijiazhuang 050011, China; 5 Pilot National Laboratory for Marine Science and Technology (Qingdao), Qingdao 266237, China; 6 Collaborative Innovation Center of Seafood Deep Processing, Dalian Polytechnic University, Dalian 116034, China

**Keywords:** domoic acid, shellfish, liquid chromatography–tandem mass spectrometry (LC–MS/MS), risk assessment, China

## Abstract

Harmful algal blooms in Chinese waters have caused serious domoic acid (DA) contamination in shellfish. Although shellfish are at particular risk of dietary exposure to DA, there have been no systematic DA risk assessments in Chinese coastal waters. A total of 451 shellfish samples were collected from March to November 2020. The presence of DA and four of its isomers were detected using liquid chromatography–tandem mass spectrometry. The spatial-temporal distribution of DA occurrence and its potential health risks were examined. DA was detected in 198 shellfish samples (43.90%), with a maximum level of 942.86 μg/kg. DA was recorded in all 14 shellfish species tested and Pacific oysters (*Crassostrea gigas*) showed the highest average DA concentration (82.36 μg/kg). The DA concentrations in shellfish showed distinct spatial-temporal variations, with significantly higher levels of occurrence in autumn than in summer and spring (*p* < 0.01), and particularly high occurrence in Guangdong and Fujian Provinces. The detection rates and maximum concentrations of the four DA isomers were low. While *C. gigas* from Guangdong Province in September showed the highest levels of DA contamination, the risk to human consumers was low. This study improves our understanding of the potential risk of shellfish exposure to DA-residues.

## 1. Introduction

Over the last 40 years, reports of harmful algal blooms (HABs) have increased significantly, particularly those involving toxic microalgae [1,2]. *Pseudo-nitzschia* spp. are pennate chain-forming diatoms that are widely distributed along coastlines worldwide, and many of them have been reported in HABs [3]. During the past two decades, there has been a rapid increase in the number of newly recorded *Pseudo-nitzschia* species and 58 species are currently recognized worldwide, 27 of which have been shown to produce domoic acid (DA) [3,4,5]. DA is toxic because its structure is similar to glutamic acid, giving it a high affinity for the propanoic acid receptors and kainate subclasses of glutamate receptors in vital organs, especially the central nervous system [6]. Widespread DA toxic incidents have occurred globally [7] and have attracted attention around the world. Canada set the first safety limit standard (20,000 μg/kg) for DA in shellfish after its first poisoning incident, and this has subsequently been adopted by other countries and international organizations, e.g., in Japan, the USA, and the European Union [8]. 

DA contamination in different shellfish is very common worldwide. The world’s first confirmed report of a DA toxic incident was the 1987 North American outbreak on Prince Edward Island due to the consumption of *Mytilus edulis* contaminated with high DA levels [9]. In 2009 in Chile, *Mesodesma donacium* was also found to contain DA [10]. DA contamination has also been reported in both wild and cultivated shellfish in many European countries, such as: *M. edulis galloprovincialis* and *Donax trunculus* in 1999, in France [11]; *Cerastoderma edule* and *Venerupis pullastra* in 2000, in Portugal [12]; *M. galloprovincialis* in 2000–2004, in Italy [13]; *M. edulis* and *Pecten maximus* in 2001–2022, in Scotland [14]; *M. galloprovincialis* and *Venus verucosa* in 2002, in Greece [15]; *P. maximus* in 2003–2004 [16], *Callista chione* and *D. trunculus* in 2008–2011 [17], in Spain; and *M. galloprovincialis* in 2011, in Croatia [18]. In Asia, the prevalence of DA contamination has also been reported in: *M. galloprovincialis* in 2018–2019, in Turkey [19]; *Perna viridis*, *Crassostrea lugubris*, and *Pinctada fucata* in 2012–2013, in Thailand [20]; and *M. trossulus* and *Mizuhopecten yessoensis* in 2009–2010, in Russia [21]. A survey in Angola showed DA contamination in *Dosinia orbignyi* Dunker, *V. corrugate* Gmelin and *Mactra glabrata* L. in 2007 [22]. In New Zealand, DA was first detected in *P. novaezealandiae* after the implementation of a comprehensive biotoxin monitoring program in early 1993 [23].

Studies have shown that different shellfish accumulate different levels of DA in their tissues [20,24], probably due to different accumulation and elimination rates and differences in the distribution of DA in the various tissues [25]. In many bivalves, most of the DA accumulates in the digestive gland, which is the main site of metabolic elimination [26,27,28,29]. It has been reported that the razor clam (*Siliqua patula*) can retain DA for an extended period and as much as one year has been recorded in the natural environments [30]. Mussels (*M. galloprovincialis*) can eliminate DA more quickly [31], while the king scallop (*P. maximus*) eliminates it more slowly [32,33]. Differences in the speed of DA elimination also occur between different species belonging to the same family. In the scallop (*Argopecten purpuratus*), DA can be rapidly transferred from the digestive gland to other organs [26]. In addition, some DA isomers have been found in shellfish, such as isoA, isoD, isoE and 5′-epimer [26,34], which exhibit different toxicities [35,36].

In recent years, DA has been routinely found in samples of Chinese shellfish, including clams, mussels, and scallops, as well as in crabs and other species [37,38,39,40,41]. *Pseudo-nitzschia* spp. are widely distributed along the coast of China, and 31 *Pseudo-nitzschia* species have been recorded [4]. Many of these have been shown to be toxigenic for DA, such as *P. simulans* sp. nov., *P. bipertita*, *P. caciantha*, *P. cuspidata*, *P. fraudulenta*, *P. fukuyoi*, *P. lundholmiae*, and *P. multiseries* [5,37,42]. Shellfish production in China is growing rapidly as a means of producing high quality protein-rich food. According to the China Fishery Statistical Yearbook, China’s aquacultural shellfish production reached 15.46 million tons in 2021 [43]. Consequently, it is vital that shellfish meant for human consumption do not contain excessive toxins. A measurable reduction in memory function was observed in Native Americans as a result of repeated dietary exposure to low levels of DA [44,45]. Chronic low level DA exposure can cause significant spatial learning impairment and hyperactivity in mice, without any visible histopathological brain lesions [46]. As the frequency of HABs in China rapidly increases, especially those caused by *Pseudo-nitzschia* spp., any contamination with DA and its subsequent accumulation in shellfish urgently requires more attention [42,47,48]. 

Dietary intake is an important means of assessing human exposure to pollutants such as polybrominated diphenyl ethers [49], ochratoxin A [50], methylmercury [51], and paralytic shellfish toxins [52]. While there are as yet no reports of human DA poisoning as a result of shellfish consumption in China, the status of contamination with DA and its isomers in different shellfish species and the potential health risks posed by exposure to DA are very poorly known and only a few Chinese investigations exist [37,38,39,40,41]. To our knowledge, this is the first study to systematically investigate and appraise human dietary exposure to DA through shellfish consumption on the coast of China. We determined the concentrations of DA and its isomers in shellfish samples using liquid chromatography–tandem mass spectrometry (LC–MS/MS) to: (1) compare DA accumulation in different shellfish species; (2) analyze the spatial-temporal distribution of DA in shellfish samples; and (3) evaluate the health risks to humans of dietary exposure to DA.

## 2. Results

### 2.1. Concentrations of DA and Its Isomers in Shellfish Samples 

A total of 451 shellfish samples collected from Chinese coastal waters were analyzed for total DA levels using LC–MS/MS. The concentration levels and detection rates for DA in different shellfish groups are presented in Table 1. Overall, DA occurred in all five groups of shellfish studied with a detection frequency of 43.90%, indicating widespread DA contamination in Chinese coastal waters. The highest detection frequency was 63.57% in oyster samples and reached more than 30% in the other four shellfish groups. The mean DA concentration in all the shellfish samples was 20.91 μg/kg, with values ranging from 0–942.86 μg/kg. The average DA concentration in oysters and scallops was higher than in the other three shellfish groups, being 49.15 and 27.97 μg/kg, respectively. The shellfish species with the highest accumulated DA concentration was also an oyster, namely *C. gigas* (942.86 μg/kg).

**Figure 1:**
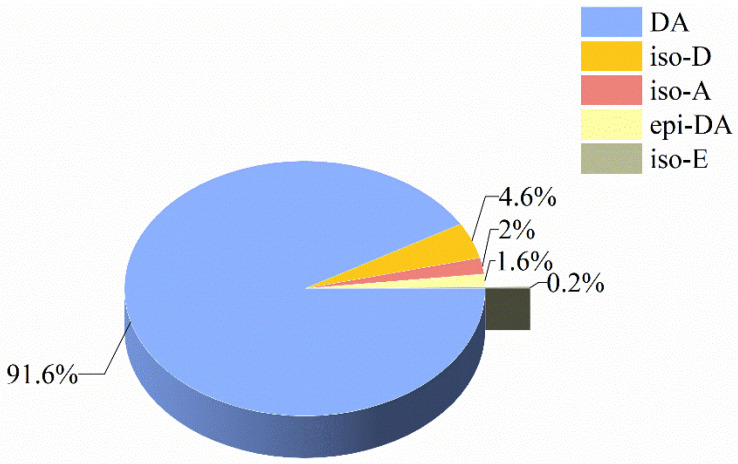
Percentage of DA and its isomers in all the shellfish samples.

In this study, we detected DA isomers in samples with total DA concentrations higher than the Limit of Detection (LOD). A typical LC–MS/MS chromatogram of DA and its isomers in standard solution and oyster samples is shown in Figure S1. The detection rates of the four DA isomers in all the shellfish samples collected were isoE (8.43%), isoD (11.31%), isoA (10.86%), and 5′-epimer (8.87%). Although the results showed that the detection rate of the different isomers was low, DA and its four isomers could be detected simultaneously in some shellfish species, e.g., *C. gigas*, *S. constricta*, and *C. hongkongensis*. However, no isomers were detected in *M. meretrix*, *M. veneriformis*, and *M. mercenaria* because their concentrations were below the LOD. The maximum isoD (42.51 μg/kg) and isoA (21.44 μg/kg) concentrations in shellfish samples were higher than for 5′-epimer DA (15.94 μg/kg) and isoE (3.55 μg/kg). The DA component alone accounted for 91% of DA and its four isomers in total in all the shellfish samples, while the concentration of isoE was less than 1% (Figure 1). 

### 2.2. Comparison of DA Concentrations in Different Shellfish Species

**Figure 2:**
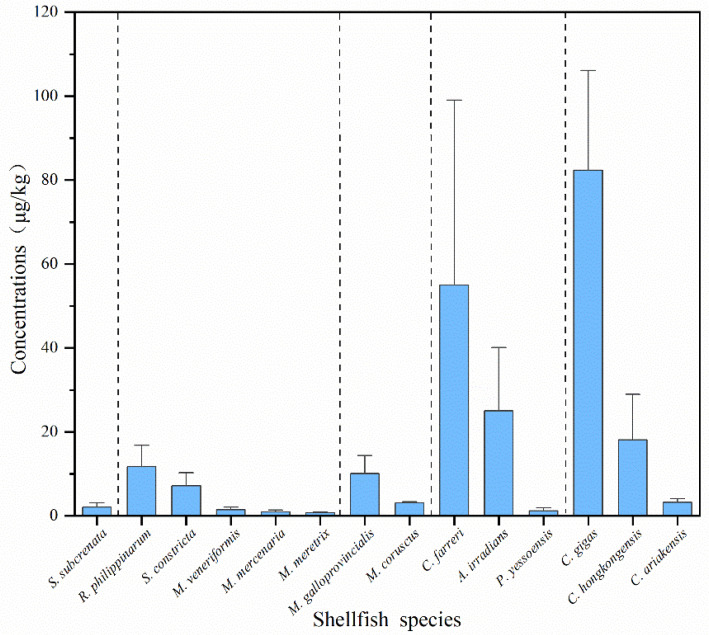
Average concentrations of DA in different shellfish species. The dotted vertical lines separate, from left to right ark shells, clams, mussels, scallops and oysters.

The results showed that the concentration of DA in different shellfish species varied greatly (Figure 2). Fourteen shellfish species were collected for DA concentration determination in this study. The maximum DA concentrations were found in *C. gigas* samples (942.86 μg/kg), followed by *C. farreri* (523.09 μg/kg). Lower concentrations were found in *A. irradians*, *M. galloprovincialis*, *C. hongkongensis*, *R. philippinarum*, and *S. constricta*. The highest average DA concentration was found in *C. gigas* (82.36 μg/kg), and the lowest in *M. meretrix* (0.71 μg/kg).

### 2.3. Spatial and Temporal Variations in DA Concentrations in Shellfish Samples

**Figure 3:**
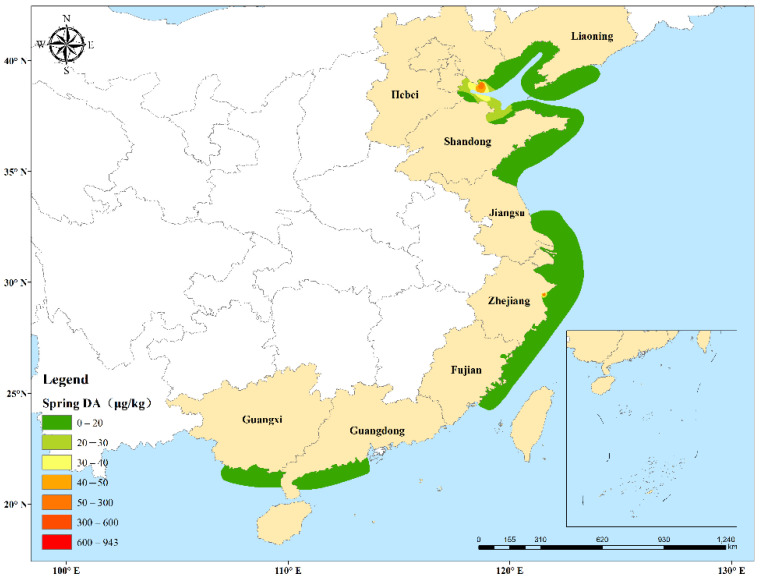

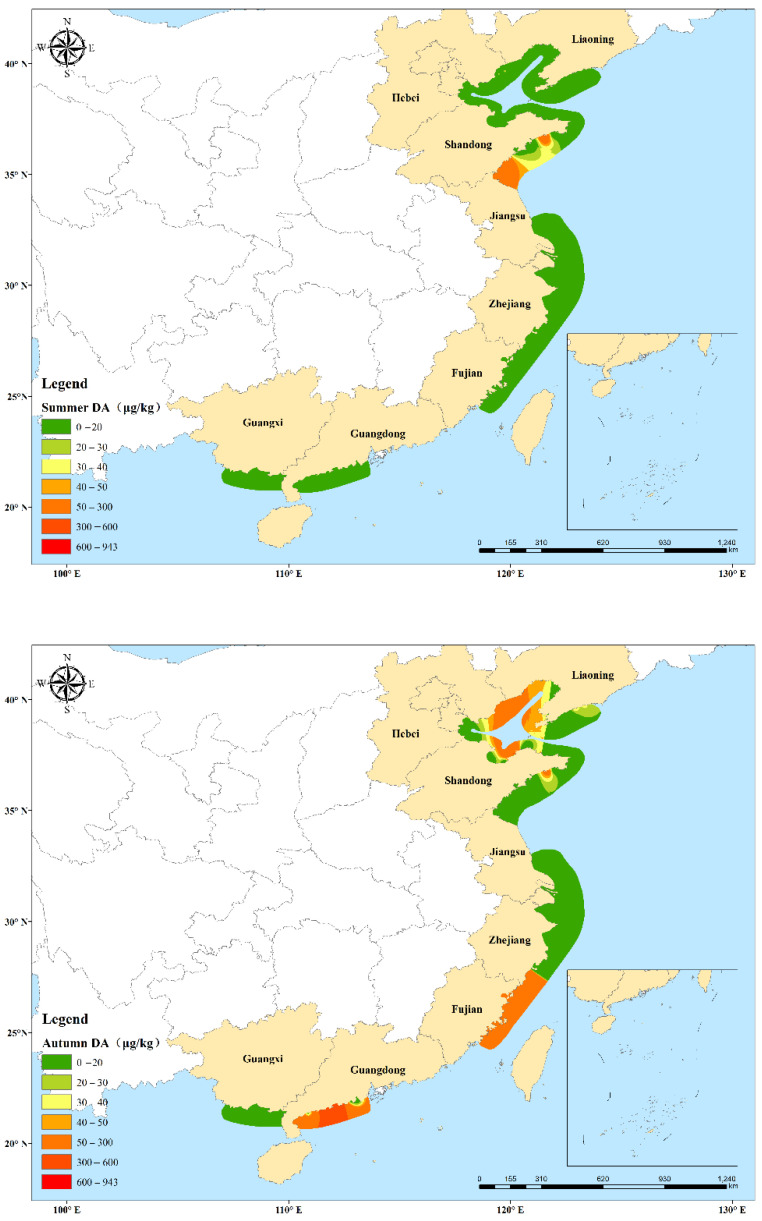
Seasonal variation of DA concentration in shellfish in the different Chinese coastal provinces surveyed.

**Figure 4:**
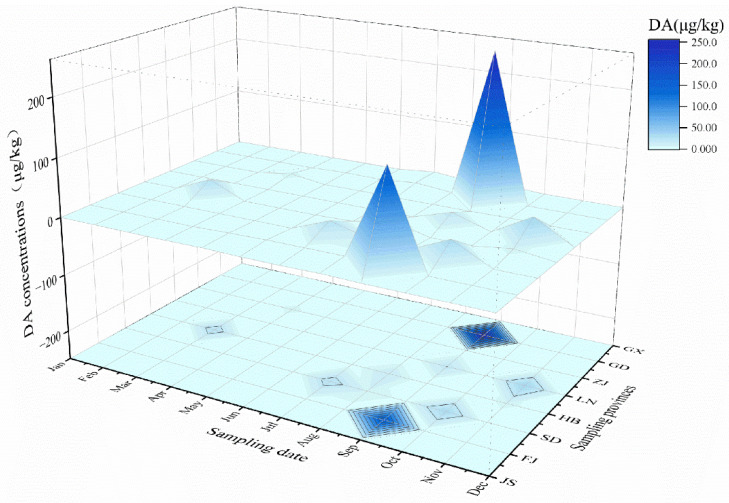
Spatial-temporal distribution of DA concentrations in shellfish sampled in the different Chinese coastal provinces surveyed. Liaoning (LN), Hebei (HB), Shandong (SD), Jiangsu (JS), Zhejiang (ZJ), Fujian (FJ), Guangdong (GD), and Guangxi (GX) Provinces. The conical shapes represent the average concentrations of DA.

A total of 128, 205, and 118 shellfish samples were collected from Chinese coastal waters in spring (March–May), summer (June–August), and autumn (September–November), respectively. Autumn had the highest DA detection rate (56.78%), followed by summer (41.95%) and spring (35.16%). The DA concentrations in autumn (average 67.67 μg/kg) were significantly higher than in summer (4.81 μg/kg) and spring (3.59 μg/kg) (*p* < 0.01), which were not significantly different to each other (*p* = 0.056). Based on the variation of the mean concentrations and detection rates of DA in shellfish, the trend during 2020 was for shellfish to accumulate higher DA concentrations from spring to autumn (Figure 3). High DA concentrations and detection rates occurred from July–October in Fujian and Guangdong Provinces, and the highest concentrations (232.82 and 942.86 μg/kg, respectively) both occurred in September (Figure 4). However, in Hebei and Shandong Provinces, DA mainly appeared in March, July, August, and October. Trace amounts of DA were detected in Liaoning, Jiangsu, Zhejiang, and Guangxi Provinces.

### 2.4. Assessment of Dietary Exposure

Total dietary exposures to DA based on estimated daily intake (EDI) and hazard quotients (HQ) for adult male and female humans are shown in Table 2. The daily aquatic food consumption of different age/gender groups ranged from 21.8 to 81.6 g/d (Table S1). The EDI and HQ values were calculated using the mean, P50, P95, and maximum concentrations of DA. The EDIs of DA in males were slightly higher than those of females in the six age groups, except for those 20–50 years-old. The mean EDIs of DA in males (0.0114 µg/kg bw/day) was lower than for females (0.0132 µg/kg bw/day). In the adult male group, the EDI value first decreased and then increased, with increasing age, with a minimum value in the 13–19 year-old group. The EDI values of the female group followed the same trend. For all age and sex groups, the HQ values were <1. The mean HQ of DA in males (0.0380%) was lower than for females (0.0439%). The HQ values showed the same trend as EDI, increasing with age in the different age/gender groups.

## 3. Discussion

Despite the substantial differences in DA accumulation, the concentrations in the five groups of shellfish samples did not exceed the 20,000 μg/kg regulatory level standard. DA was also present in 36.00% of European oysters, Queen scallops and ascidian samples in the northern Adriatic Sea, where DA concentrations ranged from 0–810 μg/kg [25]. In Lebanese coastal waters, at the same latitude as China, DA concentrations ranged from 150–3880 μg/kg in different shellfish samples [53]. DA was found at greater concentrations in cockles (840–3710 μg/kg) collected from the northwest coast of France in March to May 2010, while various concentrations were found in mussel species (720–1180 μg/kg), oysters (450–1730 μg/kg), and carpet shell clams (590–2580 μg/kg) [54]. A total of 31 of the 58 *Pseudo-nitzschia* species have been reported as widely distributed in Chinese coastal waters, and many were DA toxin-producing species [4,5,37,42]. Because of their filter feeding habits, shellfish easily accumulate DA as they filter species producing DA toxins.

So far, 11 DA derivatives have been identified in samples of naturally occurring shellfish, microalgae, and macroalgae [55]. In this study, we found DA and four of its isomers in different shellfish samples in which DA accounted for 91% of the occurrences. In scallops *A. purpuratus* collected from Bahía Tongoy in Chile, DA was the main component detected (about 90%), while the proportions of the isomers isoD, isoA, and 5′-epimer were very low [26]. In the digestive glands of mussel samples purchased north of Lisbon, Portugal, the percentages of isoD, isoA, and 5′-epimer were only 2.6%, 2.8% and 0.8%, respectively [12]. IsoD, isoE, and 5′-epimer were also present in scallop samples from the Galician Rías, Spain, after a bloom of *Pseudo-nitzschia australis* [34].

Studies have shown that different *Pseudo-nitzschia* spp. can produce different DA isomers [3]. IsoA and isoB were produced by *P. seriata* from the west coast of Greenland [56]. IsoA and isoB were detected along with DA in *P. multiseries* and *P. delicatissima*, and these isomers made up 5–6% and 4–12% of the total DA, respectively [57]. Furthermore, isoD, isoA, and 5′-epimer were also present in plankton samples collected from Luanda Bay, Angola, and Portugal [12,22], and *P. australis* and *P. multiseries* collected from sites around Aotearoa New Zealand [58]. Shellfish can accumulate different isomers in their tissues by filtering different toxic algae. There have been no previous reports of *Pseudo-nitzschia* spp. that could produce isomers in China. Photochemical transformations play important roles in natural processes, including the transformation of biotoxins in the ocean [59]. Three products, isoD, isoE, and isoF, have been identified as geometric isomers of DA produced in the photochemical degradation of DA in the ocean [60] and this may be a source of certain DA isomers found in shellfish.

Some studies have reported on the molecular mechanisms of DA elimination in shellfish. DA is a hydrophilic molecule with three carboxyl groups (pKa: 1.85, 4.47, 4.75) and an amine group (pKa: 10.60) [61,62]. It has been found that DA was present mostly in soluble form in the cytosol of the digestive gland of *P. maximus* [63]. The long retention time of DA in *P. maximus* may be due to it becoming trapped in autophagosomes [64], or due to a lack of efficient membrane transporters [63]. The transcriptional response of mussels *M. galloprovicialis* exposed to DA toxin-producing *Pseudo-nitzschia* indicated that several membrane transporters belonging to the solute carrier transporter (SLC) family were overexpressed, and that some SLCs may be related to the metabolic elimination of DA [65]. However, the reason for the high concentration of DA in razor clams (*S. patula*) may be due to the presence of more than one subtype of glutamate receptor [66]. 

The metabolic mechanism of DA elimination in oysters has been less well-studied. It has been reported that the oysters *C. virginica* eliminated DA quickly [28], but we found that *C. gigas* accumulation of DA was higher than other species in the present study. This may be related to the actual oyster species concerned. Studies have shown that the accumulation and metabolism of DA by different scallop species were significantly different [26,32]. *C. gigas* with high DA accumulations were mainly collected in September in Guangdong Province, where the abundance of DA toxin-producing *Pseudo-nitzschia* spp. was higher at this time [5,67].

*Pseudo-nitzschia* is very common in Chinese coastal waters. Thus far, studies have generally focused on southeastern Chinese coastal waters, including those of Fujian and Guangdong Provinces [4]. Twenty-nine strains belonging to seven species of *Pseudo-nitzschia* have been reported in the coastal waters of Guangdong Province, of which most could produce DA, including *P. cuspidata* and *P. multiseries* [5]. Fourteen taxa of *Pseudo-nitzschia* have been recognized in the coastal waters of Fujian Province, and three species, *P. multiseries*, *P. pseudodelicatissima*, and *P. lundholmiae*, have shown production of DA [42]. In addition, a DA toxin-producing *P. multiseries* strain was also isolated in Qingdao, Shandong Province [68]. 

In China, HABs caused by *Pseudo-nitzschia* spp. may occur throughout the year, especially in summer and autumn [48]. Of the seasonal changes, temperature is a key factor influencing the phytoplankton community structure [69,70], and can also significantly affect *Pseudo-nitzschia* growth [71,72]. *Pseudo-nitzschia* spp. have a wide range of suitable growth temperatures (from 5–32 °C), but the optimum temperature for growth in culture is more than 20 °C, especially above 25 °C [73]. In the South China Sea (including off Guangdong and Fujian Provinces), seawater temperatures in autumn range from 22–32 °C [74], suitable for the growth of *Pseudo-nitzschia* spp. *Pseudo-nitzschia cuspidata* was the main source of DA in phytoplankton samples along the coast of Guangdong Province, where seawater temperatures ranged from 24.74–30.11 °C [75]. An investigation in Daya Bay, on the coast of Guangdong Province, observed a high cell density of *Pseudo-nitzschia* (more than 6.0 × 10^5^ cells/Net Tow) during August and November 2013, with the highest DA concentration in phytoplankton samples of 2671 ng/Net Tow in September [67]. In this study, we also found the highest DA concentration in shellfish samples from Guangdong Province in September.

The Acute Reference Dose (ARfD) and Tolerable Daily Intake (TDI) are commonly used for the assessment of acute and chronic exposure to dietary toxins [76]. The acute exposure to DA in shellfish is assessed by comparing dietary exposure to DA with the ARfD. In 2009, the European Food Safety Authority (EFSA) proposed a provisional ARfD of 30 µg/kg of DA for a person weighing 60 kg [77]. As shown in Table 2, the highest acute dietary exposure value found in this study was 0.5799 µg/kg⋅bw/day in the female group (52-fold lower than the ARfD), which was higher than the 0.5020 µg/kg⋅bw/day found in the male group (60 fold lower than the ARfD). The acute dietary exposure values of the other age groups were also much lower than the 30 µg/kg⋅bw/day ARfD standard. Due to insufficient data on the chronic effects of DA, no reference for the TDI has so far been established by the EFSA or the FAO/IOC/WHO Expert Consultation panel [76]. Despite this lack of a DA TDI estimate, either by reliable authorities or formal organizations, a value of 75 µg/kg has proven to be a satisfactory working TDI value [78]. The results of this study showed that the daily DA intake of the shellfish analyzed was at least 130-fold lower than this working TDI. However, in Belgium, 5–6% of the population suffered chronic exposure to DA in scallops and exceeded the working 75 µg/kg TDI value, using a medium bound approach [79].

Different human sex and age groups have different sensitivities to DA. In old-aged rats, impaired renal clearance contributed to increased DA sensitivity [80]. With increasing age, the gastrointestinal epithelium becomes thinner and more fragile, increasing the incidence of ulcers which could enhance susceptibility to toxins [81]. This study found that elderly people (>65 years) had higher HQs than most younger age groups, in both males and females. When exposed to low levels of DA, male rats were more susceptible to severe neurotoxicity than females, although females were affected more quickly [82]. Considering the different impact of DA on different population groups, more attention should be paid to fish farmers and islanders, whose dietary intake of aquatic foods is higher than the general population [83].

## 4. Conclusions

This study assessed the dietary exposure to DA contamination in 14 shellfish species, belonging to five shellfish groups, along the coast of China. We found that oysters (especially *C. gigas*) and scallops had higher average concentrations of DA than mussels, clams, and ark shells. Four DA isomers (isoA, isoD, isoE and 5′-epimer DA) were also detected, although their detection rate and concentrations were very low in the different shellfish species studied. The DA concentration in shellfish was significantly higher in autumn than in summer and spring (*p* < 0.01) and showed obvious seasonal correlations. The highest concentrations and detection rates of DA occurred in Guangdong and Fujian Provinces, and the highest DA concentration (942.86 μg/kg) was found in the *C. gigas* samples collected from Guangdong Province in September. While the *C. gigas* samples collected from Guangdong Province in September presented the highest risk to human health, the assessment of acute and chronic exposure due to dietary DA intake indicated only a low risk to consumers.

## 5. Materials and Methods

### 5.1. Chemical and Reagents

Formic acid (≥98.0%) and ammonium formate (≥97.0%) were purchased from Merck (Darmstadt, Germany). HPLC-grade methanol and acetonitrile were provided by Fisher Scientific (Waltham, MA, USA). The water used (18.2 MΩ cm) was deionized using a Milli-Q system equipped with ion exchange and carbon filters (Millipore, Bedford, MA, USA). Bond Elut SAX cartridges (3 mL, 500 mg) were purchased from Agilent Technologies (Santa Clara, CA, USA). The 0.22 μm mixed cellulose filtration membranes were supplied by Agela Technologies (Tianjin, China). The DA calibration solution, certified reference material (CRM-DA-f: DA, 100.7 μg/mL; isoA, 0.52 μg/mL; isoD, 0.41 μg/mL; isoE, 0.013 μg/mL; and 5′-epimer, 1.1 μg/mL) was obtained from the Certified Reference Materials Program of the National Research Council (Halifax, NS, Canada). Individual stock solutions of DA were prepared in acetonitrile/water (1:9, *v*/*v*) and then stored in amber glass vials at −20 °C. Further dilutions and mixtures were made by appropriate dilution.

### 5.2. Sample Collection

**Figure 5:**
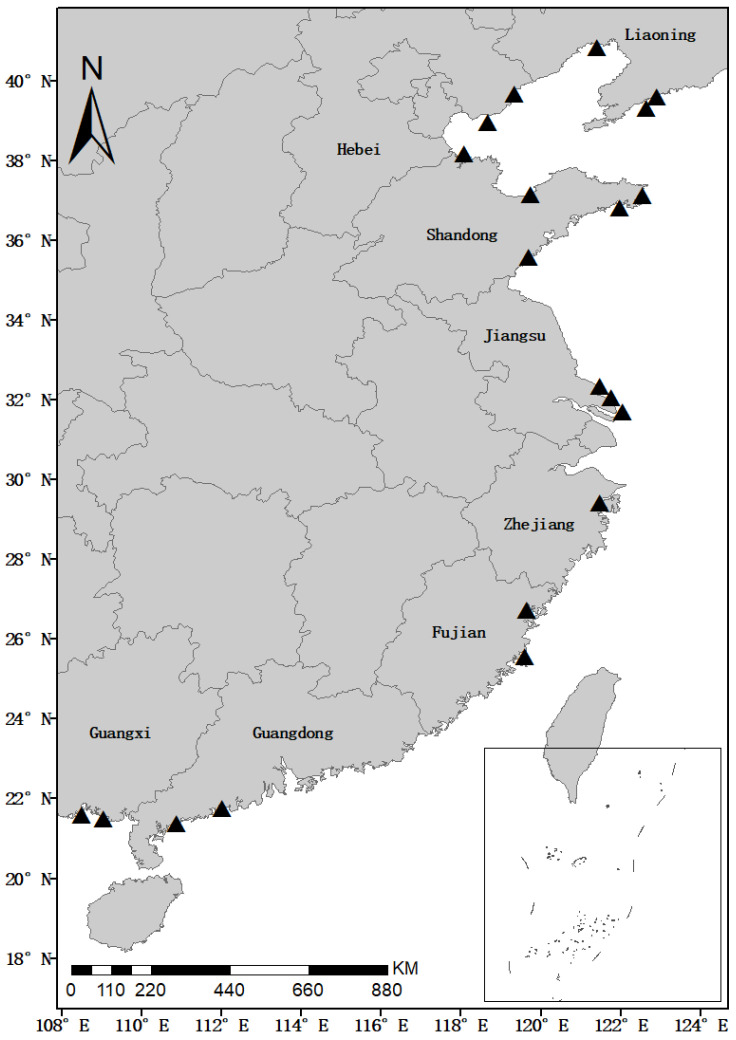
Sampling stations along the coast of China in 2020.

Shellfish samples were collected from March to November 2020 from coastal aquaculture farms in the following provinces along the coastal areas of China: Liaoning (LN), Hebei (HB), Shandong (SD), Jiangsu (JS), Zhejiang (ZJ), Fujian (FJ), Guangdong (GD), and Guangxi (GX) Provinces (Figure 5). This study collected a total of 451 diverse shellfish samples, including 192 clams (*Ruditapes philippinarum*, *Mercenaria mercenaria*, *Meretrix meretrix*, *M. veneriformis* and *Sinonovacula constricta*), 41 scallops (*A. irradians*, *Patinopecten yessoensis*, and *Chlamys farreri*), 78 mussels (*M. coruscus* and *M. edulis*), 129 oysters (*C. gigas*, *C. ariakensis*, and *C. hongkongensis*), and 11 ark shells (*Scapharca subcrenata*). All shellfish samples were collected at random from the local shellfish culture areas, washed with clean water, kept in portable icy incubators below 4 °C, and transported to the lab within 24 h. All shellfish samples were homogenized at 24,000 r/min using a T18 basic Ultra-Turrax mixer (IKA, Königswinter, Germany), and stored at −20 °C before LC–MS/MS analysis.

### 5.3. DA Extraction

The methodology for analyzing DA in shellfish samples followed previous work, with minor modifications [84]. Briefly, 5.00 ± 0.02 g samples of homogenized shellfish tissue (wet weight) were placed into 50 mL polypropylene centrifuge tubes. After addition of 12.0 mL of 50% methanol, the mixtures were vortexed for 1 min, ultrasonicated for 10 min, vortexed for 1 min, and then centrifuged at 4000 rpm for 10 min. Then, two repeat samples were extracted with 5.0 mL of 50% methanol. Both extracts were transferred to a new polypropylene tube and adjusted to 25.0 mL with the extracting solvent. Finally, the extraction solution was vortexed for 1 min and then centrifuged for 15 min at 10,000 rpm.

A total of 5.0 mL of the supernatant was transferred into a pre-activated Bond Elut SAX cartridge for purification. After that, the cartridge was washed with 5.0 mL of 10% *v*/*v* acetonitrile and eluted with 4.0 mL of 0.3% *v*/*v* formic acid. The eluate was adjusted to 4 mL with eluting solvent and then filtered through a 0.22 μm mixed cellulose filtration membrane prior to analysis by LC–MS/MS.

### 5.4. LC–MS/MS Analysis

LC–MS/MS analysis was conducted using a U3000 high performance liquid chromatography system (Thermo Scientific, Waltham, MA, USA) coupled with a triple quadrupole mass spectrometer (TSQ Endura, Thermo Scientific, Waltham, MA, USA). A Kinetex C18 column (2.1 × 100 mm, 2.6 µm, Phenomenex, Torrance, CA, USA) was used in the LC system. The injection volume of sample extracts was 10 μL. The column temperature was maintained at 35 °C. We chose a fast gradient method to initially screen the shellfish samples for total DA contamination, before the detection of isomers. The LOD was 0.08 μg/kg. The conditions of the chromatograph were as follows: mobile phase A was 2 mmol/L ammonium formate in H_2_O; mobile phase B was 100% methanol; and the fast gradient procedure flow rate was 0.30 mL/min. The composition varied as follows: 0–1 min, 20% B; 1–3 min, 20–90% B; 3.0–3.1 min, 90–20% B; and 3.1–8.0 min, 20% B. To detect the isomers, the conditions of the chromatograph were as follows: mobile phase A was 100% H_2_O, and mobile phase B was 100% acetonitrile, both of which contained 0.1% formic acid; and the slow gradient procedure flow rate was 0.30 mL/min. The composition varied as follows: 0–1 min, 5% B; 1–20 min, 5–20% B; 20–22 min, 20–5% B; and 22–25 min, 5% B. 

The mass system was equipped with an ESI source operating in positive ion mode. Under the multiple reaction monitoring mode, *m/z* 312.0 > 266.0 (quantification), *m/z* 312.0 > 248.0, and 312.0 > 161.0 (confirmation) were monitored for DA and its isomers. For the MS/MS data acquisition: the RF lens was 142 V; the dwell time was 100 ms; the collision-induced dissociation gas was 1.5 mTorr; the spray voltage was 3500 V; the sheath gas was 25 arb; the auxiliary gas was 15 arb; the ion transfer tube temperature was 300 °C; and the vaporizer temperature was 250 °C. A typical chromatogram of DA standard solution (10.0 ng/mL) and oyster samples (14.6 ng/mL) with the fast gradient method is shown in Figure S2.

### 5.5. Dietary Risk Assessment

The daily per capita intake of DA in China was calculated using the EDI (μg/kg bw/day) method [85,86,87].

The EDI was calculated as follows:

(1) $$\mathrm{EDI}=\frac{\mathrm{average}\;\mathrm{concentration}\;\mathrm{of}\;\mathrm{DA}\times \mathrm{average}\;\mathrm{daily}\;\mathrm{seafood}\;\mathrm{consumption}\;}{\mathrm{average}\;\mathrm{body}\;\mathrm{weight}}$$

The data for the average daily consumption of seafood was obtained from the 5th Chinese Total Diet Study and was 36.5 g/d [88]. An average body weight of 66.2 and 57.3 kg was used for adult males and adult females, respectively, and were calculated from the Monitoring Report on Nutrition and Health Status of Chinese Residents (2010–2013) [89].

The HQ of DA was calculated using the following equation:

(2) $$\mathrm{HQ}=\frac{\mathrm{EDI}}{\mathrm{RfD}}$$

where the oral reference dose (RfD) (µg/kg⋅bw/day) for DA is the ARfD of 30 µg/kg⋅bw/day set by the EFSA [77]. An HQ < 1.0 indicates an acceptable risk and HQ > 1.0 represents a level of exposure to DA which poses a potential health risk [50]. 

### 5.6. Statistical Analysis

Statistical analyses were performed using Microsoft Office Excel 2016 and PASW Statistics 18 (SPSS Inc., Chicago, IL, USA). The total DA concentrations reported in Table 1 and relevant Figure 2, Figure 3 and Figure 4 are calculated from the sum of all isomers. According to the relevant EFSA scientific opinion [77], the human exposure part only calculated the sum of DA+epiDA. DA concentrations in the three seasons were compared using the Mann–Whitney U test. Statistical significance was set at *p* < 0.05 and highly significant differences at *p* < 0.01. Data were represented as mean ± standard error (SE).

## Figures and Tables

**Table 1:** DA concentrations in shellfish samples.

Species	N	(%) ^a^	Mean ± SE ^b^	Min–Max ^c^
Ark shells	11	36.36	2.02 ± 1.05	0.00–9.42
Clams	192	33.85	6.24 ± 1.87	0.00–206.35
Mussels	78	43.59	9.26 ± 3.78	0.00–257.80
Scallops	41	31.71	27.97 ± 14.60	0.00–523.09
Oysters	129	63.57	49.15 ± 13.41	0.00–942.86
Total	451	43.90	20.91 ± 4.27	0.00–942.86

N: number of shellfish samples; ^a^ Values > the limit of detection (LOD); ^b^ all shellfish samples; ^c^ μg/kg.

**Table 2:** Risk assessment of DA accumulation in humans based on estimated daily intake (EDI) and hazard quotient (HQ) values for adult males and females.

	Male		Female	
	EDI	HQ (%)	EDI	HQ (%)
Mean	0.0114	0.0380	0.0132	0.0439
P50	0.0000	0.0000	0.0000	0.0000
P95	0.0583	0.1943	0.0673	0.2245
Max	0.5020	1.6732	0.5799	1.9331
Age (2–7 years) ^(a), (b)^	0.0239	0.0795	0.0239	0.0795
Age (8–12 years) ^(a), (b)^	0.0220	0.0733	0.0220	0.0733
Age (13–19 years) ^(b)^	0.0199	0.0664	0.0169	0.0563
Age (20–50 years) ^(b)^	0.0213	0.0709	0.0217	0.0723
Age (51–65years) ^(b)^	0.0235	0.0784	0.0222	0.0740
Age (>65years) ^(b)^	0.0280	0.0932	0.0235	0.0783

^(a)^ Does not distinguish sex. ^(b)^ See Table S1 for weight and daily seafood consumption data of the different age and sex groups.

## Data Availability

Not applicable.

## Supplementary Materials

The following supporting information can be downloaded at: https://www.mdpi.com/article/10.3390/toxins14120862/s1, Figure S1: Chromatogram of DA and its isomers standard solution (a) and oyster samples (b); Figure S2: Chromatogram of DA standard solution (a) and oyster samples (b).; Table S1: Daily aquatic food consumption for different age and gender groups.
