# Supplementary material for: Residue Analysis and Assessment of the Risk of Dietary Exposure to Domoic Acid in Shellfish from the Coastal Areas of China
Source: Toxins (Basel). 2022 Dec 8;14(12):862. doi: 10.3390/toxins14120862 (PMC9783215; doi:10.3390/toxins14120862)
Supplement: Supplementary file 1 [file toxins-14-00862-s001.zip › toxins-2048120-supplementary.pdf]

Supplementary Information

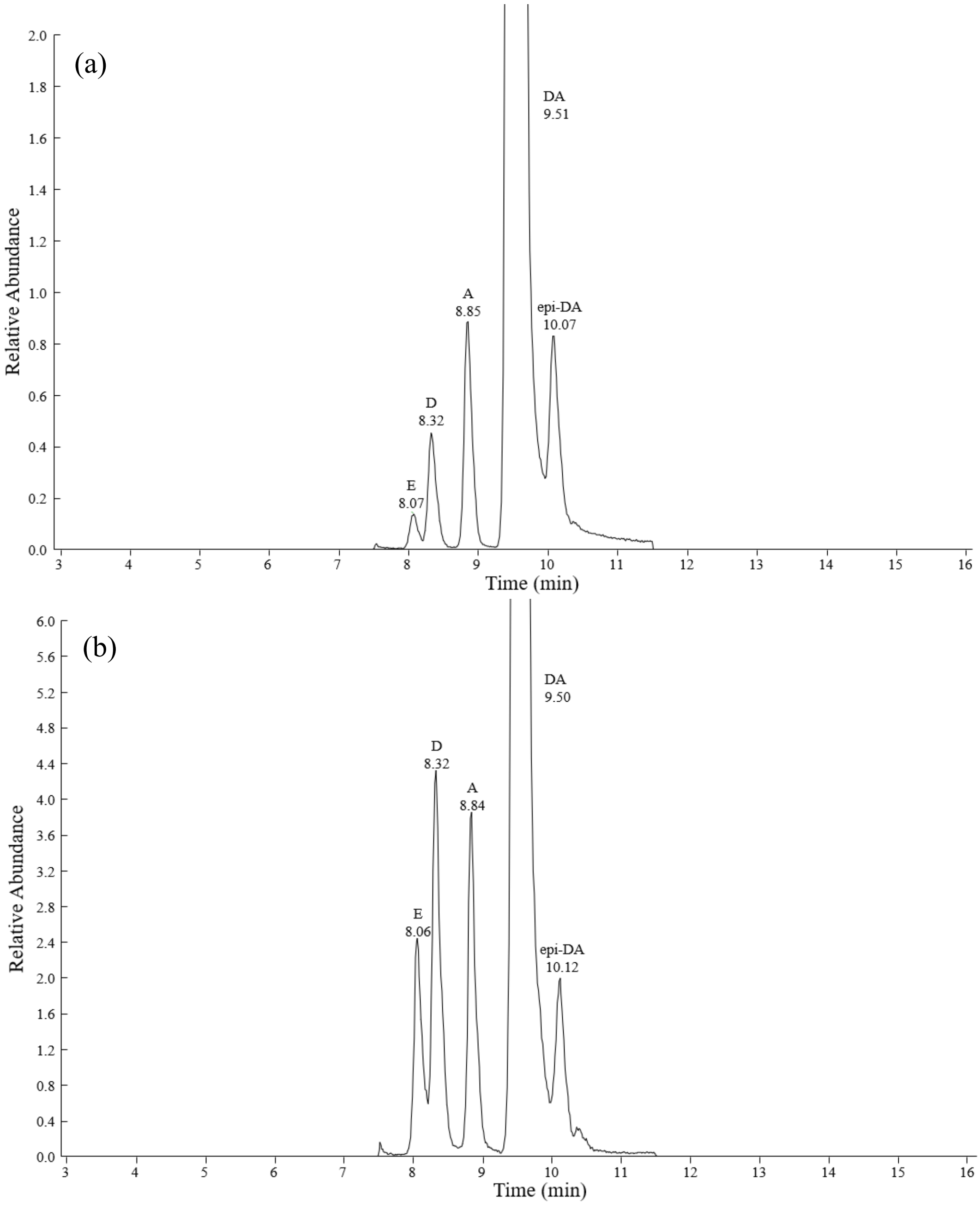

Figure S1. Chromatogram of DA and its isomers standard solution (a) and oyster samples (b).

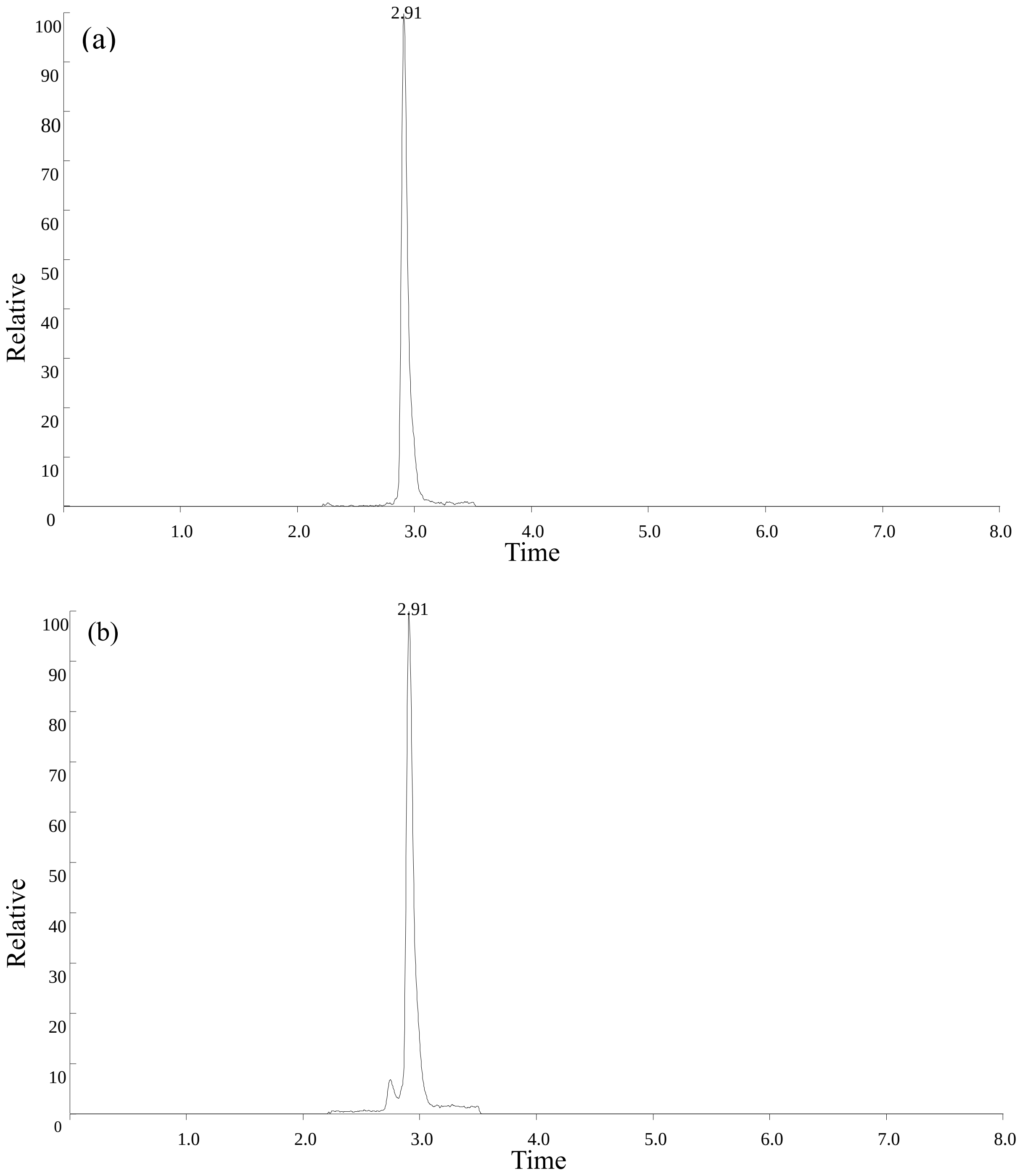

Figure S2. Chromatogram of DA standard solution 10.0 ng/mL (a) and oyster samples 14.6 ng/mL (b).

Table S1. Daily aquatic food consumption for different age and gender groups.

Age	2–7	8-12	13–19		20–50		51–65		＞65	
Gender	M/F	M/F	M	F	M	F	M	F	M	F
Weight[a] (kg)	18.9	35.1	56.2	50.6	67.4	57.3	65.5	58.8	60.4	53.7
Daily consumption[b] (g/d)	21.8	37.3	54.1	41.3	69.3	60.1	74.5	63.1	81.6	61.0

M=male; F=female; [a] data from the *Monitoring Report on Nutrition and Health Status of Chinese Residents (2010-2013)* [1]; [b] data from *The Fifth China Total Diet Study* [2].

References

1. Piao, J.H.; Huo, J.S. The monitoring report on nutrition and health status of Chinese residents (2010-2013) (Ⅱ). *People's Medical Publishing House, Beijing* **2019**; pp. 54-55.
2. Wu, Y N; Zhao,Y F.; Li, J G. *The Fifth China Total Diet Study*. Science Press, Beijing, **2018**; pp. 68.
